# Age Matters: Community Assembly in the Pig Fecal Microbiome in the First Month of Life

**DOI:** 10.3389/fmicb.2021.564408

**Published:** 2021-03-11

**Authors:** Stephanie D. Jurburg, Alex Bossers

**Affiliations:** ^1^German Centre for Integrative Biodiversity Research iDiv (Halle/Jena/Leipzig), Leipzig, Germany; ^2^Department of Infection Biology, Wageningen Bioveterinary Research, Lelystad, Netherlands; ^3^Institute of Biology, Leipzig University, Leipzig, Germany; ^4^Institute for Risk Assessment Sciences, Utrecht University, Utrecht, Netherlands

**Keywords:** pig, primary succession, fecal microbiome, community assembly, 16S rRNA gene

## Abstract

Despite the wealth of research into strategies for microbiome modulation, studies of microbiome management in pig hosts have found mixed results. A refined understanding of the patterns of microbiome assembly during the host’s early life, when management strategies are most commonly applied, is necessary for the development of successful management practices. Here, we study the development of the pig gut microbial community in a monitoring experiment, sampling the microbiome of pigs in a commercial farm intensively during the first month of life. We found that the community’s taxonomic richness increased linearly with host age. Furthermore, rapid changes across communities occurred in stages, and non-linear patterns in relative abundance were commonly observed among dominant taxa across host age, consistent with primary succession. Our results highlight the importance of understanding the patterns of microbiome assembly during host development, and identify successional stages as windows of opportunity for future research.

## Introduction

Recent advances in high throughput sequencing technologies have revealed that complex microbial communities are ubiquitous, and play an important role in the hosts they inhabit (Shreiner et al., 2015; van de Guchte et al., 2018). The assembly of gut microbial communities is essential to the development of all animals, from fish (Zhang et al., 2018; Lokesh et al., 2019), to primates, including humans (McKenney et al., 2015; Guittar et al., 2019). Altered patterns of community assembly in young hosts has been associated with dysbiosis (Neuman et al., 2018) and increased susceptibility to disease (Arruda et al., 2013). Conversely, microbiome management strategies are often most successful when applied in the earlier stages of community assembly in host microbiomes (Lin et al., 2018; Wan et al., 2019; Wang et al., 2019).

Domestic pigs (*Sus scrofa*) are important agricultural resources and animal models, and understanding how their gut microbiome develops after birth is of central importance to microbiome management strategies, which to date have found mixed success (Canibe et al., 2019). The gut microbiomes of pigs undergo extensive shifts between birth and weaning, a “developmental window” of approximately 1 month (Thompson et al., 2008) during which the host microbiome is more susceptible to external influences, including the environment (Tsai et al., 2018), host diet (Salcedo et al., 2016), and management strategies. During lactation, the pig gut microbiome increases in diversity and the microbial community changes rapidly (Frese et al., 2015).

In early life, host microbiomes are likely more dynamic due to the ongoing development of the host’s immune system, as well as due to priority effects. Priority effects occur when during the colonization of an environment, the order in which taxa arrive determines how taxa affect one another, thereby having a disproportionate influence on later community assembly (Sprockett et al., 2018). In the pig microbiome, stepwise changes in community composition have been previously demonstrated (Kim et al., 2011; Frese et al., 2015; Wang et al., 2019), however, during the first days of development, animal microbiomes can exhibit drastic changes within a daily scale (i.e., in chickens; Jurburg et al., 2019), and to date the daily fluctuations in the microbiome of newborn pigs has not been studied.

To improve the success of future microbiome management strategies, a refined understanding of the patterns of community assembly in the gut microbiome of developing pigs is necessary. Here, we sampled the fecal microbiomes of pigs at a range of ages, from birth to before weaning in order to describe community assembly in the pig gut microbiome. In line with previous findings, we hypothesized (1) that taxonomic richness (at the amplicon sequence variant, or ASV level) would increase linearly with host age, (2) that the microbiomes in hosts of the same age would exhibit similar composition, (3) that across host age, shifts in the gut microbiome would occur in stages, and (4) that the relative abundances of individual taxa would exhibit non-linear dynamics with host age, with some taxa dominating the community at certain stages and then disappearing from the microbiome.

## Materials and Methods

### Sample Collection

Samples were obtained from a Dutch farm during a single farrowing cycle in September 2018, under standard farm conditions (Supplementary Material 1). Sixty piglets farrowed from 12 sows were randomly selected for sampling out of a cohort of approximately 65 sows. Each piglet was sampled once at a single time point, and for each time point, piglets from different sows were sampled to avoid litter-based biases (Supplementary Data). All sows were previously administered *Erysipelotrichia* vaccines according to routine management procedures. The piglets included 29 female and 31 male individuals; 28 were of Great Yorkshire breed, and 32 were a crossbreed between Great Yorkshire and Dutch Landrace (Supplementary Data). During the sampling period, piglets were housed in a single pig barn within individual farrowing pens (250 × 165 cm). The pens had an iron-grid flooring, and an area with a heat lamp. Throughout the duration of the experiment, the piglets were lactating exclusively, and sows were supplied with standard lactation feed and water *ad libitum* (Supplementary Material 1).

On days 1–7, 10, 14, 21, 28, and 35 after farrowing, rectal swabs were collected from five piglets from different litters using dry swabs (Tubed Sterile Dryswab, MWE, United Kingdom). Samples were stored individually (−20°C) immediately after collection for further analyses.

### DNA Extraction and 16S rRNA Gene Amplicon Sequencing

Prior to DNA extraction, each swab was immersed in 0.5 ml of PBS buffer for 15 min, and the solution was centrifuged for 5 min at 13,000 rpm. 1 ml of pellet was used for DNA extraction with the Qiagen QIAamp Fast DNA stool mini kit (Qiagen, Hilden, Germany), according to the manufacturer’s instructions, with an extra bead-beating step, and eluted in 50 μl. Extracts were checked on a 2200 Tapestation (Agilent Technologies Santa Clara, CA, United States).

Bacterial community composition in the developing pigs’ feces was assessed by sequencing the V3–4 hypervariable region of the 16S rRNA gene as in Jurburg et al. (2019). This section was first amplified by 25 cycles of PCR using the primers CVI_V3-forw CCTACGGGAGGCAGCAG and CVI_V4-rev GGACTACHVGGGTWTCT. PCR products were checked on a 2200 Tapestation, and sequencing was performed using paired-end 300 bp sequencing on a MiSeq sequencer (Illumina Inc., San Diego, CA, United States). Negative controls were used in each round of amplification to confirm the sterility of reagents, and a mock community bacterial community was included in the sequencing run as a control.

### Sequence Processing and Statistical Analyses

Sequence processing and statistical analyses were performed in R 3.4.3 (R Core Team, 2014). The 16S rRNA gene sequencing reads were quality filtered, primer/adapter trimmed, error-corrected, dereplicated, chimera-checked, and merged using the dada2 package (v.1.4.0, Callahan et al., 2016) using standard parameters (*trimLeft* = 10; *TruncLength* = 240,210); reads were assigned with the SILVA v.132 classifier (Quast et al., 2012). Statistical analyses were performed with the *phyloseq* (McMurdie and Holmes, 2013) and *vegan* (Oksanen et al., 2007) R packages. The sequenced samples had a range of 3,321–13,550 reads per sample, and all samples were standardized to 3,221 reads per sample prior to analyses (rarefy_even_depth, seed = 1). The final dataset contained 193,260 reads and 1,813 different amplicon sequence variants (ASVs). Sequences are deposited in NCBI’s Sequence Read Archive (SRA) under BioProject accession number PRJNA594837.

Prior to linear regressions, the normality of richness data was confirmed with Shapiro–Wilk tests (*p* > 0.05). Taxonomic richness was calculated as the number of different ASVs per sample, and changes in richness over host age were fitted with a linear regression. One outlier sample from day 21 was excluded from this analysis following the assessment of q-q plots. The relative abundances of taxa at each age are reported throughout the study as *mean*±*sd*. Changes in community structure in the aging host microbiome (β-diversity), were assessed with a principal coordinates analysis (PCoA) of Bray–Curtis dissimilarities between samples at the ASV level. Clusters of samples with similar community compositions were first identified by performing Ward’s clustering of the Bray–Curtis distance matrix, and confirmed by performing *adonis* tests on the clusters. The influence of time as a categorical variable was quantified with a redundancy analysis, or RDA, using the *rda* function. The amount of turnover in community composition between two time points was measured as the pairwise Bray–Curtis dissimilarity between those two time points, and Wilcoxon tests were used to evaluate differences in turnover between non-consecutive host age comparisons, to ensure the independence of comparisons. To focus on taxa which exhibited large, consistent shifts over the period studied, we first selected genera for which an ANOVA of the effect of age on relative abundances was significant (*p* < 0.001), then standardized them according to their relative abundance patterns across ages as previously recommended (Shade et al., 2013), clustered using Euclidean distances and Ward’s method, and displayed in a heatmap.

## Results

### Taxonomic Richness in the Developing Microbiome

**FIGURE 1:**
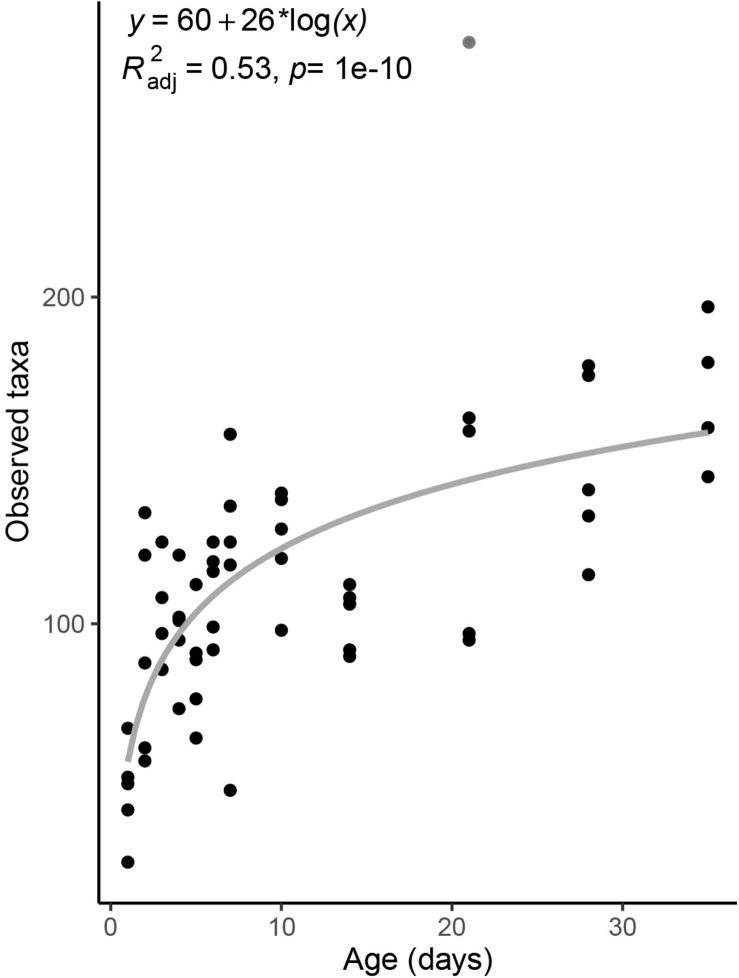
Taxonomic richness in the fecal microbiomes of pigs across age. Taxonomic richness is calculated as the total number of observed ASVs per sample. Gray line is a linear regression line. The derived formula, *R*^2^ and significance value are displayed above. One outlier from day 21 was excluded from the regression, and is shown in gray.

Taxonomic richness in the piglet fecal microbiome increased logarithmically with age, from an average of 48.4 ± 15 ASVs on day-old piglets to 168.4 ± 20 ASVs in 35 day old pigs (Figure 1). Notably, richness nearly doubled between piglets sampled one and 2 days after birth to 92.6 ± 34 ASVs, and continued to increase with host age more gradually thereafter (Figure 1).

**FIGURE 2:**
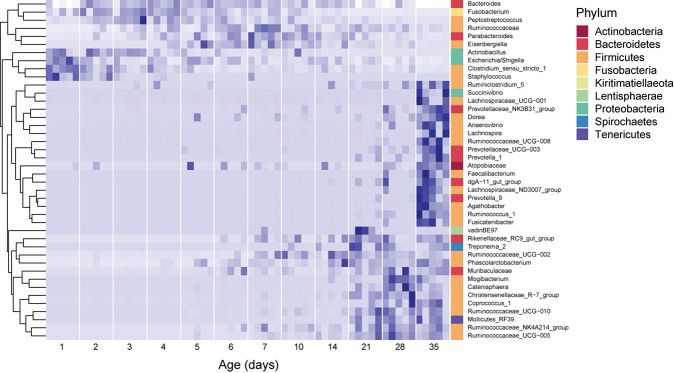
Core dynamic genera in the fecal microbiomes of pigs of different ages. The responses of 42 genera which exhibited consistent, significant changes over the period studied are displayed. For each taxon, the intensity of the blue color represents the proportion of total observations of that taxon across the whole dataset (Shade et al., 2013). These genera represent 54.2 ± 12.7% of the community, on average. Relative abundances were standardized by time, per genus, and genera were clustered according to the resulting temporal response patterns using Ward’s distance. Intensity of the blue color indicates the relative abundance of each taxon at each age group. Replicate samples are shown as columns within each age group. The lowest available classification of each genus is listed on the right, and phylum membership is indicated by the color bar on the right.

During the period studied, the fecal microbiome exhibited major compositional changes with host age as well (Figure 2). In 1 day old pigs, the microbiome was dominated by Firmicutes (42.1 ± 11.1% of the community), specifically to the genera *Clostridium sensu stricto* (20.0 ± 5.8%) and *Streptococcus* (8.1 ± 6.7%), and Proteobacteria (48.9 ± 16.9%), predominantly from the genus *Escherichia/Shigella* (44.8 ± 17.3). In 35 day old hosts, Firmicutes were still dominant (56.5 ± 5.4%), but more diverse, with *Lactobacillus* (8.4 ± 6.4%) and *Faecalibacterium* (5.1 ± 4%) exhibiting high relative abundances. The relative abundance of Proteobacteria decreased to 5 ± 2.2 for the entire phylum in 35 day old pigs. In contrast, the relative abundance of Bacteroidetes increased from 2.1 ± 3.2% on 1 day old pigs to 31.5 ± 2.9% in 35 day old pigs, and at this time was dominated by *Rikenellaceae* (8.4 ± 3.2%) and *Muribaculaceae* (5.5 ± 2.7%, Figure 2 and Supplementary Figure 2).

Surprisingly, several dominant taxa exhibited non-linear patterns with host age, increasing and decreasing in relative abundance across the 35 days of study (Figure 2). In particular, the phylum Fusobacteria (represented almost entirely by the genus *Fusobacterium*) was dominant in hosts between 2 and 4 days old (13.9 ± 6.5% of the community), but exhibited much lower relative abundances in pigs of all other ages (3.4 ± 4.1% of the community for all other samples). Similarly, the genus *Lachnoclostridium* (Firmicutes) exhibited higher relative abundances in hosts between 2 and 28 days old (6.3 ± 5.1% of the community), and were much lower in hosts that were 1 or 35 days old (0.1 ± 0.4%). Furthermore, we observed several taxa which were not dominant in the community, but occurred in all the animals of specific ages. In 1 day old hosts, *Actinobacillus* and *Staphylococcus* were present in all samples, but only represented 0.08 ± 0.5 and 0.1 ± 0.4 % of the community, respectively.

### Community Structure and Community Turnover

**FIGURE 3:**
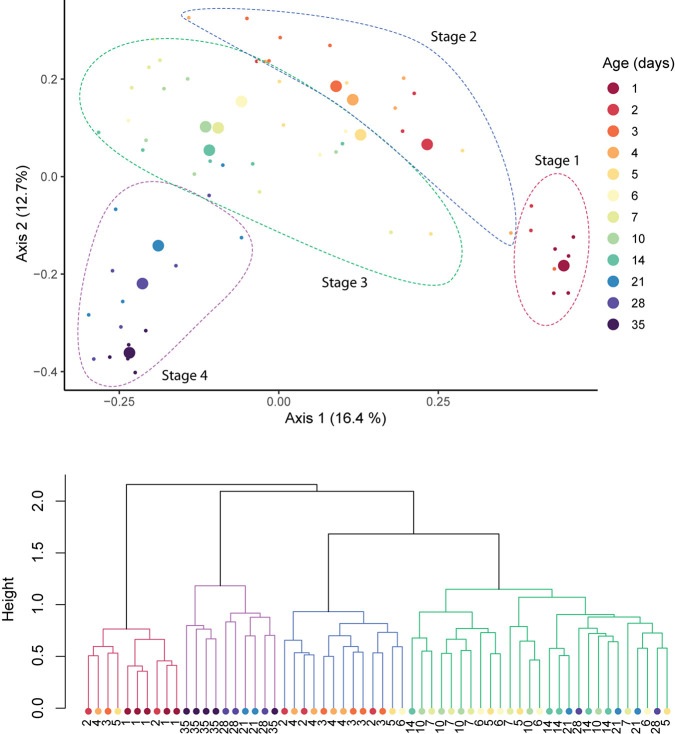
Sequential shifts in the community structure of the fecal microbiomes of pigs after birth. A PCoA plot of Bray–Curtis distances between samples (top). Large circles represent the centroid of all samples belonging to pigs of the same age, and small circles indicate individual samples. Clustering of samples was confirmed with *adonis* (*p* = 0.001) and clusters are outlined with dotted lines. Ward’s clustering of Bray–Curtis distances (bottom). Clusters are indicated by branch color.

Time explained 21.83% of the variance in community composition (RDA, *p* = 0.01). No effect of host breed, sex, or litter on the composition of the fecal microbiomes was found (Supplementary Table 1), and were thus excluded from further statistical models. Across the period studied, the microbial community underwent sequential shifts, with the fecal microbiome increasingly diverging from its original (i.e., 1 day old pigs) conformation with host age (Figure 3, top). Clustering analysis revealed four clusters of samples, which were driven by host age (Figure 3, bottom). Clusters were significantly different from each other (adonis; *R*^2^ = 0.089, *p* = 0.001; Figure 3), and were dominated by samples from 1-day, 2–4-day, 5–21-day, and 28–35-day-old hosts.

**FIGURE 4:**
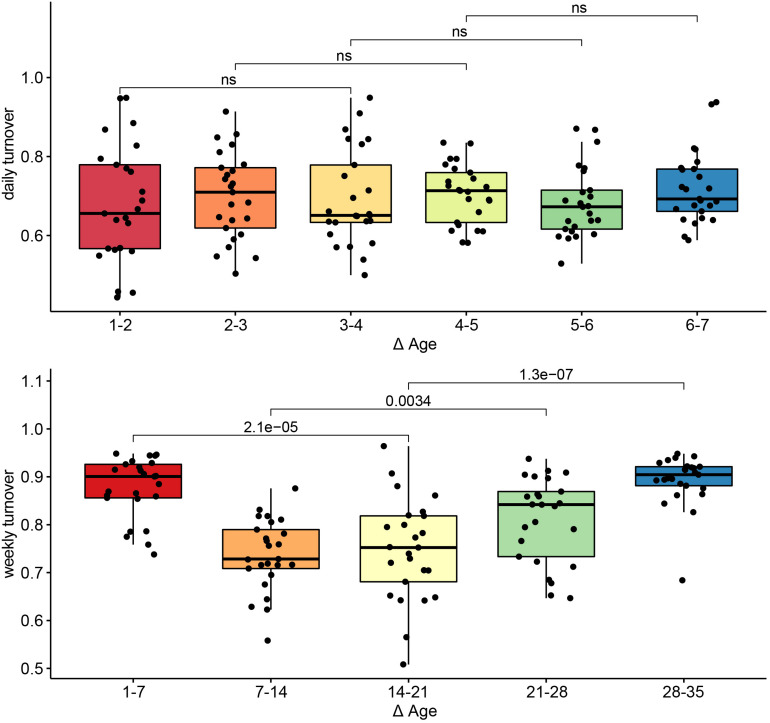
Turnover in community structure during primary succession. Pairwise Bray–Curtis distances in community structure between samples taken on consecutive days (top) or consecutive weeks (bottom). Differences in turnover between non-consecutive comparisons were assessed with Wilcoxon tests, and the *p*-values for each comparison are indicated. Comparisons for which *p* > 0.05 are labeled *ns*, or non-significant.

To assess turnover in the host’s fecal microbiome through aging, we looked at the pairwise dissimilarity in community composition between consecutive ages at both daily (days 1–7) and weekly (weeks 1–5) intervals. During the first week of the experiment, turnover was temporally stable (*p* > 0.05 for all Wilcoxon tests, Figure 4, left). At a weekly interval, however, turnover changed with host age (*p* < 0.01 for all Wilcoxon tests), exhibiting the highest mean turnover between 1 and 7 days of age and the lowest turnover between 7 and 14 days of age, increasing gradually thereafter (Figure 4, right).

## Discussion

Host health, growth, behavior, and the resident bacterial community are tightly linked (Theriot and Young, 2015; Neuman et al., 2018; Guevarra et al., 2019). Animal microbiomes are most susceptible to change during the host’s early life (Thompson et al., 2008). However, the microbiome of young hosts develops in concert with the host’s physiology, and the assembly of the microbiome over time is rarely integrated into extant management strategies (Canibe et al., 2019; Guevarra et al., 2019). Here, we sampled the fecal microbiomes of pigs during the first month of life at a range of ages in order to (1) determine whether the microbiome increases in taxonomic complexity with host age, (2) determine whether shifts in microbiome composition after birth are consistent across animals, (3) resolve the timing of these shifts, and (4) identify the dominant taxa at each stage. We used the fecal microbiome as a proxy for the successional dynamics in the gastrointestinal tract of the pigs (Frese et al., 2015; Mach et al., 2015).

### Patterns of Microbial Diversity in the Developing Pig Microbiome

We found that the fecal microbiomes of piglets undergo extensive changes during the first 48 h after birth. Microbial community diversity was nearly twice as high in 2-day old pigs as in 1-day old pigs. However, relative to 2 day old pigs, the diversity in 3 day old pigs was only 8.4% higher, on average. The rapid growth in microbial diversity in the pig microbiome has been previously documented (Kim et al., 2011; Frese et al., 2015), however, our findings show that most of this increase is logarithmic and happens during the first 2 days. Similar patterns have been found for the chicken microbiome after hatching (Jurburg et al., 2019; Kers et al., 2019).

### Successional Stages in the Developing Pig Gut Microbiome

We found clear successional stages in the developing gut microbiome. Turnover in the community was highest during the first week of sampling, and decreased thereafter. This gradual stabilization of the microbial community has been previously observed in pigs, as well as in other animals (Thompson et al., 2008; Videnska et al., 2014; Costea et al., 2017; Zou et al., 2020), however, this is the first study to employ a daily sampling during the first week of life. Every sample was taken from a different animal, in order to exclude the disproportionate influence of specific individuals, and to better capture between-animal variability. Nevertheless, we detected consistent changes in community composition and community diversity across animals, with host age. These changes were independent of the sow or farrowing pen, suggesting that they resulted from the host’s development. Future studies to determine the universality of these successional patterns are necessary.

We focused on development before weaning, which is often targeted for management (Thompson et al., 2008). We therefore also excluded the influence of the dietary switch on the microbiome, but it is likely that weaning would have strongly affected the pig’s gastrointestinal microbiome thereafter (Lallès et al., 2007; Frese et al., 2015; Mach et al., 2015; Ke et al., 2019). We identified four stages: Stage 1 (day 1), Stage 2 (days 2–4), Stage 3 (days 5–21), and Stage 4 (days 28–35). Our sampling focused on ages of highest expected variation. The statistically significant clustering of samples from hosts of 5–21 days of age into a single group suggests that the stages we detected were not biased by this sampling scheme.

Shifts between stages were associated with the gradual appearance of certain taxa: the shift between Stage 1 and Stage 2 was characterized by a decrease in the relative abundance of the populations of *Clostridium sensu stricto* and *Escherichia/Shigella*, and the increase in the relative abundances of *Fusobacterium, Lachnospiraceae, Lachnoclostridum, and Lactobacillus*, as well as members of the phylum Bacteroides. Interestingly, the increased prevalence of *Fusobacterium* has been associated with colorectal cancer (Kelly et al., 2018), malnutrition (Alou et al., 2019), and impaired immune recovery in humans (Lee et al., 2018), and dysbiosis in suckling pigs (Huang et al., 2019). In our study, however, *Fusobacterium* gradually decreased with host age in the absence of treatment, and was no longer dominant in 21-day-old hosts. Conversely, *Lachnospiraceae* has been associated with the suppression of *Clostridium difficile* infections (Reeves et al., 2012).

The shift between Stage 2 and Stage 3 was characterized by the decrease in *Fusobacterium* and *Clostridium sensu stricto*, the transient further increase in the relative abundance of *Lactobacillus*, and the appearance of *Rikenellaceae*, and *Treponema*. These three taxa have been observed to increase in response to feed supplements or prebiotics (Dicksved et al., 2015; Fan et al., 2017; Roselli et al., 2017). Finally, the shift between Stage 3 and Stage 4 was characterized by the dominance of generally beneficial gut microbes *Lachnospiraceae, Lactobacillus*, and *Rikenellaceae*, as well as increases in several members of the family Ruminococcaceae.

The sampling of pigs at daily age intervals provided deeper insights into the successional patterns exhibited by the fecal microbiome of piglets, from birth to weaning. Our results highlight the need to consider temporal dynamics into microbiome management frameworks. We observed non-linear dynamics in several taxa including *Fusobacterium*, *Peptostreptococcus, Parabacteroides*, and *Eisenbergiella*, which have been associated with gut dysbiosis (Wang et al., 2012; Bao et al., 2018; Huang et al., 2019). By the end of the experiment, however, these taxa’s relative abundances had dramatically decreased or become undetectable. Previous studies have similarly found *Fusobacterium* in pigs to decrease in relative abundance naturally after 25 days (Ke et al., 2019). Without sufficient temporal samples, these non-linear patterns may be perceived as linear increases or decreases, or may be missed entirely. During periods of dominance, these taxa reached abundances above 1% of the community for several or all of the animals, suggesting that their role in the succession is important and may play a role on the outcome of management regimes.

### Leveraging Successional Dynamics as Windows of Opportunity for Management

The experimental setup employed in this study (i.e., sampling different animals in each age group) reveals, first, that successional dynamics occur similarly across animals of a single farming system, regardless of the identity of the parent or the immediate environment (i.e., farrowing pen). This suggests that management applied at a single time point will target the microbiomes of all animals at the same successional stage, at least within a single farming system. Further studies across more diverse farming systems are necessary to determine the generalizability of these successional stages. Secondly, each successional stage is characterized by the dominance of a specific taxon or group of taxa. This highlights the need to integrate knowledge of successional dynamics into the development of microbiome management frameworks, and consider which successional stage is the target for modification through management. Because specific taxa are present at each stage, the success of management is likely time-dependent. Thirdly, bacterial taxa which have been associated with negative physiological outcomes are present in healthy hosts at different developmental stages, adding further nuance to research into the development of microbiome management strategies.

**FIGURE 5:**
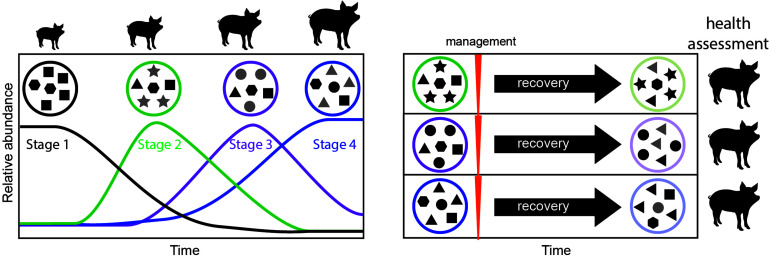
Proposed framework for evaluating windows of opportunity for management in developing animals. In a first step, the successional dynamics in the developing animal are assessed over time, to determine the timing of successional stages (left). Microbial communities in each successional stage are represented as a circle, and each shape represents a different microbial taxon. Newly dominant taxa for each stage are highlighted in gray. In a second step management is applied at each successional stage, followed by a period of recovery, and monitoring of the resulting long-term microbiome (right). To assign value or desirability to each outcome and the best timing for management, the animal’s health, and productivity (e.g., feed conversion efficiency) can be measured.

Our study underscores the need to explicitly consider time as a key factor modulating the outcome of microbiome management strategies We propose that successional stages identified here may serve as windows of opportunity for management (Figure 5). Managing the microbiomes of organisms at the same successional stage will likely yield less variable and more reliable outcomes. In contrast, targeting different successional stages for management will likely result in different outcomes, some of which may be more desirable, providing avenues for improving microbiome management practices. Future research should focus on determining the universality of the successional stages presented here, and on measuring the differential effect of applying management to each developmental window.

## Data Availability Statement

The sequence data are publicly available through the Sequence Read Archive, BioProject accession number PRJNA594837. Additional metadata is available in the Supplementary Material. Codes used in this study are available at https://github.com/drcarrot/Community-assembly-in-the-pig-microbiome.

## Ethics Statement

Directive 2010/63/EU Art. 3 and the Animal Welfare Body of Wageningen University and Research did not require the study to be reviewed or approved by an ethics committee, because each animal was swabbed a single time, causing minimal discomfort. Written informed consent was obtained from the owners for the participation of their animals in this study.

## Author Contributions

SJ designed the experiment, performed data analyses, and wrote the manuscript. AB and SJ secured funding for the project. AB was involved in project management, sequencing, and manuscript editing. Both authors contributed to the article and approved the submitted version.

## Conflict of Interest

The authors declare that the research was conducted in the absence of any commercial or financial relationships that could be construed as a potential conflict of interest.

## References

[B1] Alou M. T. Bachar D. Levasseur A. Brah S. Alhousseini D. Sokhna C. (2019). Gut microbiota alteration is characterized by a *Proteobacteria* and fusobacteria bloom in kwashiorkor and a bacteroidetes paucity in marasmus. *Sci. Rep.* 9:9084. 10.1038/s41598-019-45611-3 31235833 PMC6591176

[B2] Arruda P. H. E. E. Madson D. M. Ramirez A. Rowe E. Lizer J. T. Songer J. G. (2013). Effect of age, dose and antibiotic therapy on the development of *Clostridium difficile* infection in neonatal piglets. *Anaerobe* 22 104–110. 10.1016/j.anaerobe.2013.04.010 23624068

[B3] Bao J. Zheng H. Wang Y. Zheng X. He L. Qi W. (2018). Echinococcus granulosus infection results in an increase in *Eisenbergiella* and *Parabacteroides genera* in the gut of mice. *Front. Microbiol.* 9:2890. 10.3389/fmicb.2018.02890 30555437 PMC6281689

[B4] Callahan B. J. McMurdie P. J. Rosen M. J. Han A. W. Johnson A. J. A. Holmes S. P. (2016). DADA2: high-resolution sample inference from Illumina amplicon data. *Nat. Methods* 13:581. 10.1038/nmeth.3869 27214047 PMC4927377

[B5] Canibe N. O’Dea M. Abraham S. (2019). Potential relevance of pig gut content transplantation for production and research. *J. Anim. Sci. Biotechnol.* 10 1–19. 10.1186/s40104-019-0363-4 31304012 PMC6604143

[B6] Costea P. I. Hildebrand F. Manimozhiyan A. Bäckhed F. Blaser M. J. Bushman F. D. (2017). Enterotypes in the landscape of gut microbial community composition. *Nat. Microbiol.* 3 8–16. 10.1038/s41564-017-0072-8 29255284 PMC5832044

[B7] Dicksved J. Jansson J. K. Lindberg J. E. (2015). Fecal microbiome of growing pigs fed a cereal based diet including chicory (*Cichorium intybus* L.) or ribwort (*Plantago lanceolata* L.) forage. *J. Anim. Sci. Biotechnol.* 6:53. 10.1186/s40104-015-0054-8 26688727 PMC4683726

[B8] Fan P. Liu P. Song P. Chen X. Ma X. (2017). Moderate dietary protein restriction alters the composition of gut microbiota and improves ileal barrier function in adult pig model. *Sci. Rep.* 7:43412. 10.1038/srep43412 28252026 PMC5333114

[B9] Frese S. A. Parker K. Calvert C. C. Mills D. A. (2015). Diet shapes the gut microbiome of pigs during nursing and weaning. *Microbiome* 3 1–10. 10.1186/s40168-015-0091-8 26167280 PMC4499176

[B10] Guevarra R. B. Lee J. H. Lee S. H. Seok M.-J. Kim D. W. Kang B. N. (2019). Piglet gut microbial shifts early in life: causes and effects. *J. Anim. Sci. Biotechnol.* 10:1. 10.1186/s40104-018-0308-3 30651985 PMC6330741

[B11] Guittar J. Shade A. Litchman E. (2019). Trait-based community assembly and succession of the infant gut microbiome. *Nat. Commun.* 10 1–11. 10.1038/s41467-019-08377-w 30710083 PMC6358638

[B12] Huang A. Cai R. Wang Q. Qu W. Shi L. Li C. (2019). Dynamic change of gut microbiota during porcine epidemic diarrhea virus infection in suckling piglets. *Front. Microbiol.* 10:322. 10.3389/fmicb.2019.00322 30858839 PMC6397872

[B13] Jurburg S. D. Brouwer M. S. M. Ceccarelli D. van der Goot J. Jansman A. J. M. Bossers A. (2019). Patterns of community assembly in the developing chicken microbiome reveal rapid primary succession. *Microbiologyopen* 8:e00821. 10.1002/mbo3.821 30828985 PMC6741130

[B14] Ke S. Fang S. He M. Huang X. Yang H. Yang B. (2019). Age-based dynamic changes of phylogenetic composition and interaction networks of health pig gut microbiome feeding in a uniformed condition. *BMC Vet. Res.* 15:172. 10.1186/s12917-019-1918-5 31126262 PMC6534858

[B15] Kelly D. Yang L. Pei Z. (2018). Gut microbiota, fusobacteria, and colorectal cancer. *Diseases* 6:109. 10.3390/diseases6040109 30544946 PMC6313651

[B16] Kers J. G. Fischer E. A. J. Stegeman J. A. Smidt H. Velkers F. C. (2019). Comparison of different invasive and non-invasive methods to characterize intestinal microbiota throughout a production cycle of broiler chickens. *Microorganisms* 7:431. 10.3390/microorganisms7100431 31658673 PMC6843853

[B17] Kim H. B. Borewicz K. White B. A. Singer R. S. Sreevatsan S. Tu Z. J. (2011). Longitudinal investigation of the age-related bacterial diversity in the feces of commercial pigs. *Vet. Microbiol.* 153 124–133. 10.1016/j.vetmic.2011.05.021 21658864

[B18] Lallès J. P. Bosi P. Smidt H. Stokes C. R. (2007). Nutritional management of gut health in pigs around weaning. *Proc. Nutr. Soc.* 66 260–268. 10.1017/S0029665107005484 17466106

[B19] Lee S. C. Chua L. L. Yap S. H. Khang T. F. Leng C. Y. Azwa R. I. R. (2018). Enrichment of gut-derived Fusobacterium is associated with suboptimal immune recovery in HIV-infected individuals. *Sci. Rep.* 8:14277. 10.1038/s41598-018-32585-x 30250162 PMC6155144

[B20] Lin C. Wan J. Su Y. Zhu W. (2018). Effects of early intervention with maternal fecal microbiota and antibiotics on the gut microbiota and metabolite profiles of piglets. *Metabolites* 8:89. 10.3390/metabo8040089 30563199 PMC6316024

[B21] Lokesh J. Kiron V. Sipkema D. Fernandes J. M. O. Moum T. (2019). Succession of embryonic and the intestinal bacterial communities of Atlantic salmon (*Salmo salar*) reveals stage-specific microbial signatures. *Microbiologyopen* 8:e00672. 10.1002/mbo3.672 29897674 PMC6460355

[B22] Mach N. Berri M. Estellé J. Levenez F. Lemonnier G. Denis C. (2015). Early-life establishment of the swine gut microbiome and impact on host phenotypes. *Environ. Microbiol. Rep.* 7 554–569. 10.1111/1758-2229.12285 25727666

[B23] McKenney E. A. Rodrigo A. Yoder A. D. (2015). Patterns of gut bacterial colonization in three primate species. *PLoS One* 10:e0124618. 10.1371/journal.pone.0124618 25970595 PMC4430486

[B24] McMurdie P. J. Holmes S. (2013). phyloseq: an R package for reproducible interactive analysis and graphics of microbiome census data. *PLoS One* 8:e61217 10.1371/journal.pone.0061217. 23630581 PMC3632530

[B25] Neuman H. Forsythe P. Uzan A. Avni O. Koren O. (2018). Antibiotics in early life: dysbiosis and the damage done. *FEMS Microbiol. Rev.* 42 489–499. 10.1093/femsre/fuy018 29945240

[B26] Oksanen J. Blanchet F. G. Kindt R. Legendre P. Minchin P. R. O’Hara R. B. (2007). The vegan package. *Community Ecol.*

[B27] Quast C. Pruesse E. Yilmaz P. Gerken J. Schweer T. Yarza P. (2012). The SILVA ribosomal RNA gene database project: improved data processing and web-based tools. *Nucleic Acids Res.* 41 D590–D596. 10.1093/nar/gks1219 23193283 PMC3531112

[B28] R Core Team (2014). *R: A Language and Environment for Statistical Computing.*

[B29] Reeves A. E. Koenigsknecht M. J. Bergin I. L. Young V. B. (2012). Suppression of *Clostridium difficile* in the gastrointestinal tracts of germfree mice inoculated with a murine isolate from the family Lachnospiraceae. *Infect. Immun.* 80 3786–3794. 10.1128/IAI.00647-12 22890996 PMC3486043

[B30] Roselli M. Pieper R. Rogel-Gaillard C. de Vries H. Bailey M. Smidt H. (2017). Immunomodulating effects of probiotics for microbiota modulation, gut health and disease resistance in pigs. *Anim. Feed Sci. Technol.* 233 104–119. 10.1016/j.anifeedsci.2017.07.011

[B31] Salcedo J. Frese S. A. Mills D. A. Barile D. (2016). Characterization of porcine milk oligosaccharides during early lactation and their relation to the fecal microbiome. *J. Dairy Sci.* 99 7733–7743. 10.3168/jds.2016-10966 27522435 PMC5557353

[B32] Shade A. McManus P. S. Handelsman J. (2013). Unexpected diversity during community succession in the apple flower microbiome. *MBio* 4:e00602–12. 23443006 10.1128/mBio.00602-12 PMC3585449

[B33] Shreiner A. B. Kao J. Y. Young V. B. (2015). The gut microbiome in health and in disease. *Curr. Opin. Gastroenterol.* 31:69. 10.1097/MOG.0000000000000139 25394236 PMC4290017

[B34] Sprockett D. Fukami T. Relman D. A. (2018). Role of priority effects in the early-life assembly of the gut microbiota. *Nat. Rev. Gastroenterol. Hepatol.* 15 197–205. 10.1038/nrgastro.2017.173 29362469 PMC6813786

[B35] Theriot C. M. Young V. B. (2015). Interactions between the gastrointestinal microbiome and *Clostridium difficile*. *Annu. Rev. Microbiol.* 69 445–461. 10.1146/annurev-micro-091014-104115 26488281 PMC4892173

[B36] Thompson C. L. Wang B. Holmes A. J. (2008). The immediate environment during postnatal development has long-term impact on gut community structure in pigs. *ISME J.* 2 739–748. 10.1038/ismej.2008.29 18356821

[B37] Tsai T. Sales M. A. Kim H. Erf G. F. Vo N. Carbonero F. (2018). Isolated rearing at lactation increases gut microbial diversity and post-weaning performance in pigs. *Front. Microbiol.* 9:2889. 10.3389/fmicb.2018.02889 30555436 PMC6282802

[B38] van de Guchte M. Blottière H. M. Doré J. (2018). Humans as holobionts: implications for prevention and therapy. *Microbiome* 6:81. 10.1186/s40168-018-0466-8 29716650 PMC5928587

[B39] Videnska P. Sedlar K. Lukac M. Faldynova M. (2014). Succession and replacement of bacterial populations in the caecum of egg laying hens over their whole life. *PLoS One* 9:e115142. 10.1371/journal.pone.0115142 25501990 PMC4264878

[B40] Wan J. Lin C. Ren E. Su Y. Zhu W.-Y. (2019). Effects of early intervention with maternal faecal bacteria and antibiotics on liver metabolome and transcription in neonatal pigs. *Front. Physiol.* 10:171. 10.3389/fphys.2019.00171 30890952 PMC6413716

[B41] Wang T. Cai G. Qiu Y. Fei N. Zhang M. Pang X. (2012). Structural segregation of gut microbiota between colorectal cancer patients and healthy volunteers. *ISME J.* 6 320–329. 10.1038/ismej.2011.109 21850056 PMC3260502

[B42] Wang X. Tsai T. Deng F. Wei X. Chai J. Knapp J. (2019). Longitudinal investigation of the swine gut microbiome from birth to market reveals stage and growth performance associated bacteria. *Microbiome* 7:109. 10.1186/s40168-019-0721-7 31362781 PMC6664762

[B43] Zhang Z. Li D. Refaey M. M. Xu W. Tang R. Li L. (2018). Host age affects the development of southern catfish gut bacterial community divergent from that in the food and rearing water. *Front. Microbiol.* 9:495. 10.3389/fmicb.2018.00495 PMC5869207 29616008

[B44] Zou X. Liu G. Meng F. Hong L. Li Y. Lian Z. (2020). Exploring the rumen and cecum microbial community from fetus to adulthood in goat. *Animals* 10:1639. 10.3390/ani10091639 PMC7552217 32932976

