# Supplementary material for: Age Matters: Community Assembly in the Pig Fecal Microbiome in the First Month of Life
Source: Front Microbiol. 2021 Mar 11;12:564408. doi: 10.3389/fmicb.2021.564408 (PMC8006452; doi:10.3389/fmicb.2021.564408)
Supplement: Supplementary file 2 [file Image_1.PDF]

Supplementary Materials

S1. Supplementary methods

Sows were fed with industrially produced sow feed with the following composition: crude protein 15%, crude fat 5.6%, crude fiber 4.9%, crude ash 6.5%, lysine 0.89%, methionine 0.28%, calcium 1.02%, phosphorus 0.54%, sodium 0.27%. Ingredients included barley, corn, soy meal, bakery and pasta products, wheat semolina, wheat, wheat (decomposed), sunflower seed meal, linseed, beet molasses, calcium carbonate, soybean husks, beet pulp, monocalcium phosphate, sugar, sodium chloride, fish oil, magnesium oxide, palm oil.

The farm had a high health status according to Dutch regulations. That is, sows were tested quarterly for Aujeszky's disease virus (ADV), porcine reproductive and respiratory syndrome (PRRS), *Mycoplasma hyopneumoniae, Actinobacillus pleuroplneumoniae* (APP), *Brachyspira hyodysenteriae, Pasteurella multocida,* classical swine fever (CSF), African swine fever, *Brucella*, Methicillin-resistant *Staphylococcus aureus* (MRSA), *Salmonella*, transmissible gastroenteritis (TGE), Influenza, ectoparasites, rotavirus, *Leptospira*, porcine epidemic diarrhea (PED), and swine vesicular disease (SVD).

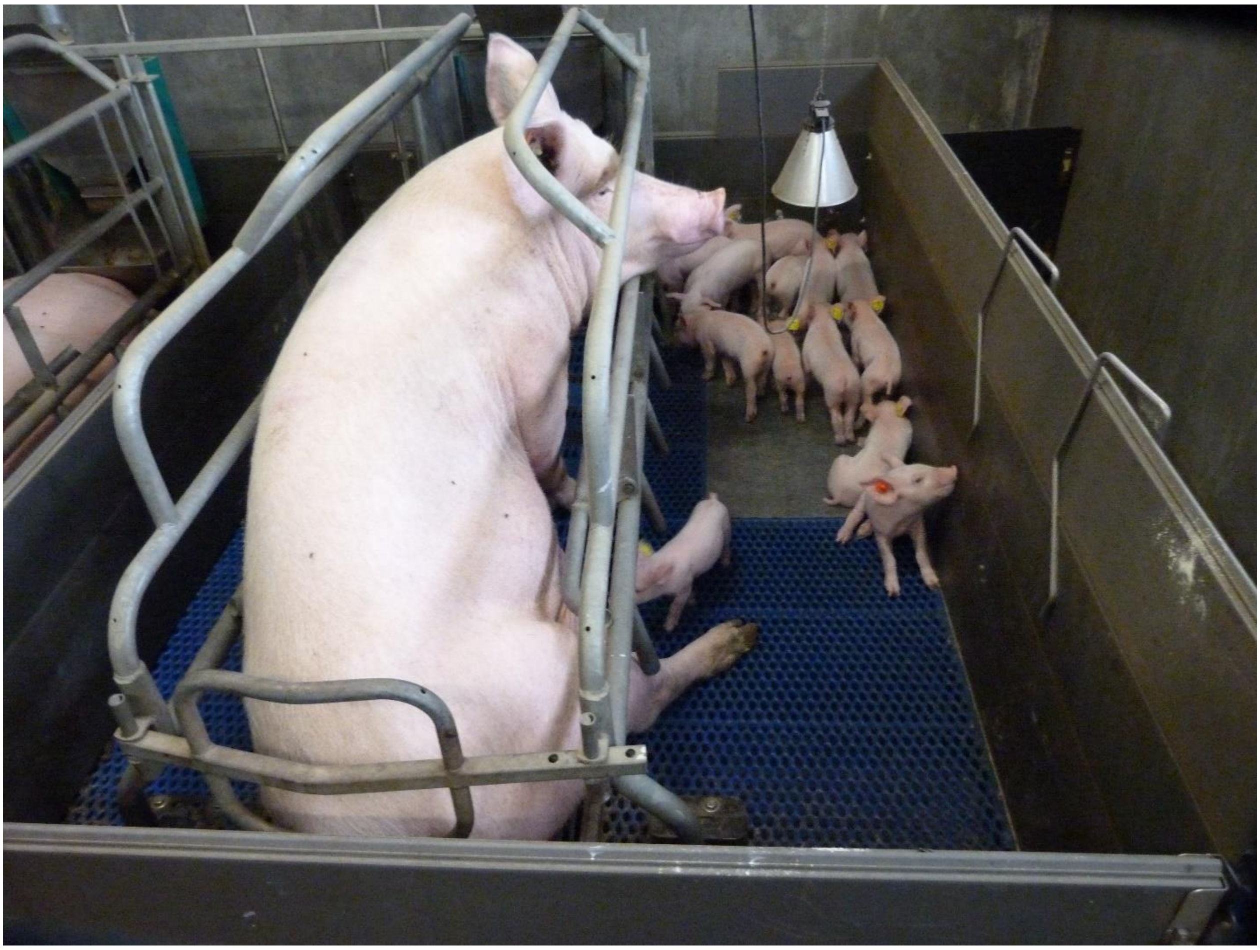

S1.1. Pens. Each sow was held in a 250 cm * 165 cm pen with its piglets. The pens had an iron-grid flooring, and an area with a heat lamp. Each pen contained one sow and its litter.

Table S1.1. Effect of random variables on the fecal microbiomes of piglets. Results of three PERMANOVA-like *Adonis* analyses of the effect of litter, sex, and breed on the Bray-Curtis distance matrices of microbiomes. Random factors had no effect on the microbiomes of piglets studied (*p*>0.34 for all comparisons). Furthermore, an RDA for the effect of Sow, Sex and Breed was not significant (p=0.96).

	**Df**	**SumOfSqs**	**R^2**	**F**	**Pr(>F)**
Sow	1	0.267	0.01343	0.7893	0.736
Residual	58	19.622	0.98657		
Total	59	19.889	1		
	**Df**	**SumOfSqs**	**R^2**	**F**	**Pr(>F)**
Sex	1	0.2278	0.01145	0.6719	0.921
Residual	58	19.6615	0.98855		
Total	59	19.8892	1		
	**Df**	**SumOfSqs**	**R^2**	**F**	**Pr(>F)**
Breed	1	0.3539	0.01779	1.0507	0.344
Residual	58	19.5353	0.98221		
Total	59	19.8892	1		

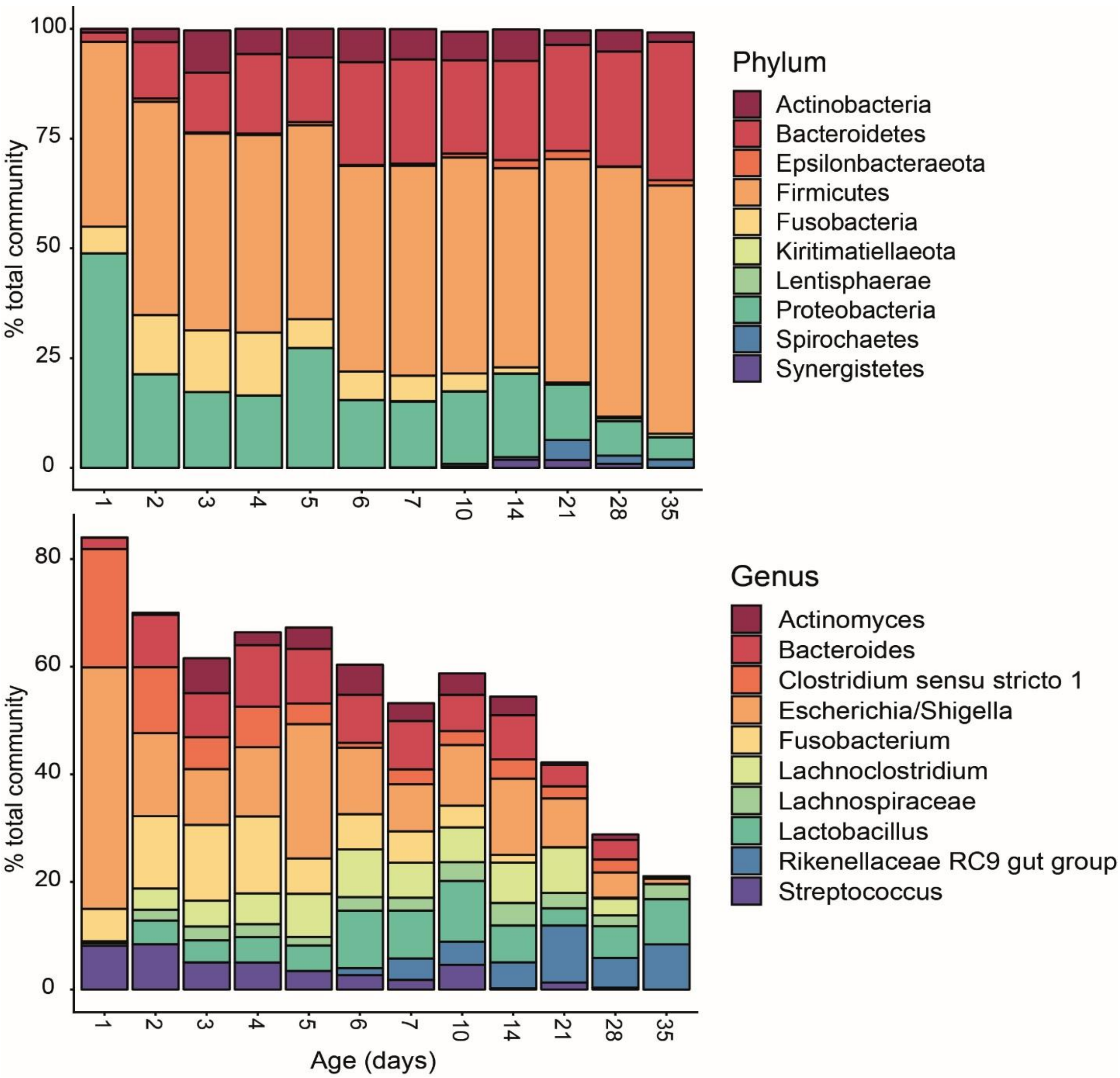

Figure S2. Patterns of dominance along primary succession. Relative abundances of the 10 most abundant phyla (top) and genera (bottom) on average over the 35 days of study. Each stacked bar depicts the average relative abundance of taxa across the 5 animals sampled at each time point. The total height of each bar indicates the proportion of the community represented by the 10 taxa.
